# Extracellular Vesicles Transfer the Receptor Programmed Death-1 in Rheumatoid Arthritis

**DOI:** 10.3389/fimmu.2017.00851

**Published:** 2017-07-24

**Authors:** Stinne R. Greisen, Yan Yan, Aida S. Hansen, Morten T. Venø, Jens R. Nyengaard, Søren K. Moestrup, Malene Hvid, Gordon J. Freeman, Jørgen Kjems, Bent Deleuran

**Affiliations:** ^1^Department of Biomedicine, Aarhus University, Aarhus, Denmark; ^2^Department of Rheumatology, Aarhus University Hospital, Aarhus, Denmark; ^3^Interdisciplinary Nanoscience Center (iNANO), Aarhus University, Aarhus, Denmark; ^4^Department of Molecular Biology and Genetics, Aarhus University, Aarhus, Denmark; ^5^Department of Clinical Medicine, Sterology and Electron Microscopy Laboratory, Centre for Stochastic Geometry and Advanced Bioimaging, Aarhus University Hospital, Aarhus, Denmark; ^6^Department of Clinical Biochemistry, Aarhus University Hospital, Aarhus, Denmark; ^7^Deparment of Clinical Medicine, Aarhus University Hospital, Aarhus, Denmark; ^8^Department of Medical Oncology, Dana-Farber Cancer Institute, Boston, MA, United States

**Keywords:** programmed death-1, extracellular vesicles, synovium, microRNA, rheumatoid arthritis

## Abstract

**Introduction:**

Extracellular vesicles (EVs) have been recognized as route of communication in the microenvironment. They transfer proteins and microRNAs (miRNAs) between cells, and possess immunoregulatory properties. However, their role in immune-mediated diseases remains to be elucidated. We hypothesized a role for EVs in the rheumatoid arthritis (RA) joint, potentially involving the development of T cell exhaustion and transfer of the co-inhibitory receptor programmed death 1 (PD-1).

**Methods:**

Synovial fluid mononuclear cells (SFMCs) and peripheral blood mononuclear cells (PBMCs) from RA patients were investigated for PD-1 and other markers of T cell inhibition. EVs were isolated from RA plasma and synovial fluid. In addition, healthy control (HC) and RA PBMCs and SFMCs were cultured to produce EVs. These were isolated and investigated by immunogold electron microscopy (EM) and also co-cultured with lymphocytes and PD-1 negative cells to investigate their functions. Finally, the miRNA expression profiles were assessed in EVs isolated from RA and HC cell cultures.

**Results:**

Cells from the RA joint expressed several T cell co-inhibitory receptors, including PD-1, TIM-3, and Tigit. ELISA demonstrated the presence of PD-1 in EVs from RA plasma and synovial fluid. Immunogold EM visualized PD-1 expression by EVs. Co-culturing lymphocytes and the PD-1 negative cell line, U937 with EVs resulted in an induction of PD-1 on these cells. Moreover, EVs from RA PBMCs increased proliferation in lymphocytes when co-cultured with these. All EVs contained miRNAs associated with PD-1 and other markers of T cell inhibition and the content was significantly lower in EVs from RA PBMCs than HC PBMCs. Stimulation of the cells increased the miRNA expression. However, EVs isolated from stimulated RA SFMCs did not change their miRNA expression profile to the same extend.

**Conclusion:**

EVs carrying both the PD-1 receptor and miRNAs associated with T cell inhibition were present in RA cell cultures. Upon stimulation, these miRNAs failed to be upregulated in EVs from RA SFMCs. This was in line with increased expression of T cell co-inhibitory markers on SFMCs. In conclusion, we suggest EVs to play a significant role in the RA microenvironment, potentially favoring the progression of T cell exhaustion.

## Introduction

A central part of many inflammatory diseases is the progression into chronicity. This process is still poorly understood but involves both pro-inflammatory and anti-inflammatory components.

T cell exhaustion is a state of cellular dysfunction that is described in infections, cancers, and autoimmune diseases (1–4). In infections and cancers, the exhausted T cells counteract the ability of the immune system to clear the infection or the tumor cells. This causes a weak and incomplete immune response and may increase spreading of pathogens or cancer cells (5). In a prolonged inflammatory response, the exhausted T cells develop into a hyporesponsive state and are, therefore, associated with the progression of chronicity (6). The function of exhausted T cells in autoimmune diseases is less clear. However, these cells have recently been suggested to be associated with a better prognosis (4). Exhausted cells are mainly described within the CD8^+^ T cell subpopulation (7), but exhausted CD4^+^ T cells (8) and B cells (9, 10) are also described in relation to chronic inflammatory diseases.

Exhaustion of both CD4^+^ and CD8^+^ T cell is characterized by expression of the co-inhibitory receptors programmed death 1 (PD-1), lymphocyte-activation gene 3, cytotoxic T-lymphocyte-associated protein 4 (CTLA-4), T-cell immunoreceptor with Ig and ITIM domains (Tigit), and T cell immunoglobulin and mucin-domain containing-3 (TIM-3). Cytokine production, especially interleukin-(IL)-2, tumor necrosis factor-α, and interferon-γ, is reduced and in addition, proliferation and motility is limited (5, 7, 11). There are multiple subsets of exhausted T cells, and depending on the expression of transcription factors, some exhausted T cells can be revived into functional T cells (12). A high expression of T-bet and a low expression of Eomes favors cells to be capable of returning to a functional state by a relevant stimulation or blocking antibodies (13, 14).

Extracellular vesicles (EVs) are emerging as important transporters in the immune system, offering a protected route of transportation of genetic material and proteins between cells (15, 16). They range in size from 50 nm to 1 µm, and may be released as exosomes from the endosomal pathway, as microvesicles by budding from the cell membrane, or as apoptotic bodies (16). Most cells produce EVs and they are found in all body fluids and cell culture supernatant (17–19). Their influence is documented in several autoimmune diseases (20), including rheumatoid arthritis (RA) (21). Besides their association with diseases, they are important communicators in the immune system with a dual function in both increasing and decreasing the immune activity of recipient cells (22).

Extracellular vesicles are known to carry small non-coding RNA sequences, known as microRNA (miRNA) (23), that can regulate the stability and translation of messengerRNA (mRNA) (24). miRNAs are considered important regulators of genes associated with immune activity and have been described in relation to cancers and autoimmune diseases (25, 26).

Although EVs are present in RA, their relation to disease is poorly understood. We hypothesized a role for EVs in the RA joint and, here, we report that EVs isolated from RA patients are transporters of the co-inhibitory receptor PD-1 and miRNAs favoring PD-1 expression in addition to other co-inhibitory receptors associated with T cell exhaustion. We suggest that EVs from RA patients transfer their cargo to cells in the microenvironment, thereby augmenting the development of chronicity in these patients.

## Materials and Methods

### Patient Material

Peripheral blood mononuclear cells (PBMCs), plasma, synovial fluid, and synovial fluid mononuclear cells (SFMCs) were obtained from patients with chronic RA at the out-patient’s clinic at Aarhus University Hospital, Denmark. All RA patients were over the age of 18 and fulfilled the American College of Rheumatology 1987 revised criteria for RA. Patients were treated in accordance with treatment guidelines for RA (27). When patients presented with disease flare, typically a swollen knee joint PBMCs and SFMCs were obtained. Cells were isolated by Ficoll-Paque PLUS (GE Healthcare) and kept at −135°C until usage. Plasma and synovial fluid was kept at −80°C. All patients gave informed written consent to participate in the study. The protocol was conducted in accordance with the Helsinki Declaration, and approved by the Danish ethics committee and the Danish data protection agency (20121329). Cells from healthy controls (HC) were obtained from buffy coats, from an established cooperation with the Danish blood bank. HCs cannot be traced or identified. These cells were treated in accordance with cells from RA patients and stored at −135°C until usage. It is not possible to collect HC SFMCs. As PBMCs and SFMCs differ significantly, comparisons are mainly made between HC and RA PBMCs and RA PBMCs and SFMCs.

### Staining for T Cells Co-Inhibitory Receptors

Peripheral blood mononuclear cells from HCs and paired PBMCs and SFMCs from RA patients were stained for the presence of T cell co-inhibitory receptors. Non-specific binding was blocked by 50 µg/ml mouse IgG (Jackson), and surface staining was performed using the following antibodies: CD3 V450 (clone: UCHT1, BD), CD4 PE-CF594 (clone: RPA-T4, BD), CD8 BV785 (clone: RPA-T8, BioLegend), CD25 Alexa 700 (clone: BC96, BioLegend), Tim-3 BV711 (clone: F38-2E2, BioLegend), CTLA-4 PerCPCy5.5 (clone: L3D10, BioLegend), Tigit PE-Cy7 (clone: MBSA43, eBioscience), PD-1 APC (clone: MIH4, BD), Live-dead near IR (Thermo Fisher Scientific). Cells were permeabilized by BD Facs lysing solution and BD Facs Perm Solution 2 (both BD bioscience). Intracellular staining was performed using Eomes PE (clone: WD1928 ebioscience). All antibodies were used in the concentration recommended by the manufacturer. Gating was done on lymphocytes, excluding doublets and dead cells. Gates were set using FMOs. CD3^+^ CD4^+^ and CD3^+^ CD8^+^ cells were investigated for their expression of T cell co-inhibitory receptors.

### An *In Vitro* Model for Repeatedly Stimulated T Cells

CD4^+^ T cells were isolated from paired PBMCs or SFMCs by negative selection using the EasySep Human CD4^+^ T cell Isolation Kit (Stemcell Technologies). All stimulations were done in duplicates. The isolated cells were directly lysed in RNA lysis buffer (Macherey-Nagel) to assess baseline transcription level, or resuspended in RPMI (Gibco) supplemented with 10% ultracentrifuged (UC) FCS (Sigma), 10 mM HEPES (Gibco) 2 mM glutaMAX (Gibco), and 2.5 nM sodium pyruvate. Repetitive stimulated T cells were generated by seeding 5 × 10^5^ isolated CD4^+^ T cells at a density of 1 × 10^6^ cells/ml in a 48-well plate pre-coated with 2 µg/ml anti-CD3 (clone OKT-3, eBioscience) and anti-CD28 (clone CD28.2, eBioscience). Following 5 days of stimulation, cells were transferred to a new uncoated 48-well plate for 10 days of resting and restimulated with anti-CD3/anti-CD28 for an additional period of 5 days. The cell culture medium was refreshed with 20 U/ml human rIL-2 (Roche Diagnostics) every third day during the entire culture period. At indicated time-points (day 5, 15, and 20), an aliquot of the cell cultures was harvested. The supernatant was collected for PD-1 ELISA (R&D systems) and the cell pellet was lysed in RNA lysis buffer.

RNA was extracted from the CD4^+^ T cells using the Nucleospin RNA Kit (Macherey-Nagel) according to manufacturer’s protocol. Twelve microliters of the extracted RNA were converted into cDNA using the QuantiTect Revers Transcription Kit (Qiagen). Prior to real-time PCR the cDNA was diluted 1:10 in RNase-free water. Real-time PCR analysis for PD-1 and FoxP3 was done using Brilliant SYBRgreen QPCR Mastermix (Agilent Technology) using primer sets from DNA Technology, Denmark: the following primer sets were used for the evaluation of PD-1 and FoxP3 (DNA Technology): PPIB fw 5′-TGTGGTGTTTGGCAAAGT and rev 5′-TGGAATGTGAGGGGAGTG; FoxP3 fw 5′-CACCTGGCTGGGAAAATGG and rev 5′-GGAGCCCTTGTCGGATGAT; and PD-1 fw 5′-GGCGGCCAGGATGGTTCTTA and rev 5′-CAGGTGAAGGTGGCGTTGT. The primers were used in a final concentration of 300 nM and the real-time PCR analysis was performed in a Stratagene 3005 Mx Pro (Agilent Technology) with the following thermal cycle: 95°C for 5 min followed by 45 cycles of 95°C for 30 s, 58°C for 30 s, and 72°C for 30 s. The expression level of FoxP3 and PD-1 was calculated relative to the reference gene PPIB using the 2^−ΔCt^ method.

### Generating and Isolating EVs

Peripheral blood mononuclear cells and SFMCs were stimulated with plate-bound anti-CD3, 1 µg/ml (clone: F7.2.38, Dako) and anti-CD28, 1 µg/ml (clone: CD28.2, BD) for 48 h in EV-free media (RPMI supplemented with: 1% penicillin/streptamycin, 1% glutamine). Non-stimulated cells were also cultured for 48 h. Cells and dead cells were excluded by two centrifugations at 335 *g* for 10 min. Cell debris were excluded by UC at 30,000 *g* for 35 min. EVs were isolated by UC at 100,000 *g* for 90 min (28). We chose this protocol to obtain a high number of vesicles.

### Capturing EVs on Beads

The Exo-flow purification kit (Cat: EXOFLOW300A-1, System Bioscience) was used to confirm the presence of EVs in plasma and synovial fluid according to manufacturer’s commercial protocol. In short, purified EVs were captured on beads using an anti-CD63 antibody. Beads with EVs and control beads were stained with a secondary FITC antibody. The supernatant after the final EV isolation was used as a negative control.

### NanoSight Nanoparticle Tracking Analysis

The generated EVs from PBMCs and SFMCs were diluted in PBS and analyzed using NanoSight LM10 (Malvern Instruments) with a 405 nm laser. Measurements were performed in five times of 60 s video captures of each sample with camera level 15 and detection threshold 10 for all analysis. The data were analyzed using software version 3.1 to determine the concentration and size of the EVs.

### ELISA

The presence of PD-1 on the EVs was measured by ELISA. EVs were isolated from plasma and synovial fluid using the commercial available ExoQuick kit (System biosciences) according to manufacturer’s instructions. This isolation method was based on polymer gradient centrifugation resulting in a lipid-soluble fraction and a water-soluble fraction. Each fraction was diluted 1:1 in PBS with 20 µg/ml goat and bovine IgG. The two fractions and the plasma/synovial fluid were examined in duplicates using a commercially available and previously validated PD-1 ELISA (R&D systems) (29).

### Electron Microscopy (EM) Staining

The EV pellet was resuspended in 200 µl 4% paraformaldehyde, 0.2% glutaraldehyde (GTA). After 10 min fixation, 10 ml PBS was added and the EVs were UC at 100,000 × *g*, 90 min, 4°C. The pellet was resuspended in 100 µl PBS. Nickel grids were mounted in 50 µm drops and rested for 25 min. 50 mM glycine in PBS was added. Blocking was performed in Aurion blocking solution for gold goat conjugates. Anti-PD-1 antibody (clone: NAT105, Abcam) was diluted 1:50 and grids were incubated o/n. Secondary 10 nm goat anti-mouse (Aurion) was diluted 1:20, and incubated for 2 h. Grids were post fixed in 2% GTA for 5 min. After the final wash, grids were contrast stained with 0.5% uranylacetate. The stained drops from EV pellets were then visualized in a FEI Morgagni 268 transmission electron microscope equipped with a SIS III digital camera.

### RNA Purification, Small RNA Library Preparation, and Sequencing

Total RNA from EVs was purified using miRCURY RNA isolation kit-Cell and Plant (Exiqon) and eluted in 100 µl RNase free water. The RNA was concentrated by ethanol precipitation done by adding 1 µl glycoblue (Ambion, Thermo Fischer Scientific), 10 µl 3 M pH 5.5 sodium acetate (Ambion, Thermo Fischer Scientific), and 250 µl pre-cooled 99% ethanol to the samples. The samples were incubated at −2°C overnight. The final RNA pellet was washed with 1 ml 75% ethanol. The RNA pellet was re-suspended in 7 µl RNase free water. The small RNA library of EVs was constructed using Illumina TruSeq Small RNA Sample Prep Kit (Illumina) by adding 5 µl of total RNA as input. Due to lower RNA input compared with the standard protocol, the amount of adaptors was reduced to 1/10. All other reagents were reduced to 1/2, and PCR cycles were increased from 12 to 15. The size and purity of the cDNA libraries were validated on a 2100 Bioanalyzer High Sensitivity DNA chip (Agilent) and the concentration was quantified using KAPA Library Quantification Kit (KAPA biosystems). The libraries were purified in the size range of 140–160 bp by Pippin Prep (Sage Science), and then pooled as required, to be sequenced on the Illumina HiSeq 2000 instrument by Beijing Genomics Institute, China.

### Sequencing Data Analysis

FASTX-Toolkit was used to trim away low-quality reads and remove adaptor sequences from raw reads. The annotation analysis was performed by mapping the clean reads to a list of datasets using Bowtie. First, miRNAs were annotated by mapping to human miRNAs and other miRNAs from miRBase v21 allowing zero mismatches. Other relevant small RNAs (piRNA, tRNA, snRNA, snoRNA, and Y RNA) were annotated allowing one mismatch. To assess degradation, the remaining unmapped reads were mapped to long RNA datasets: rRNA, other RNAs from Rfam and mRNA. The expression of miRNA was normalized by reads per million (RPM) using the formula: miRNA RPM normalized expression = (miRNA counts/the total counts of all mapped miRNAs) × 10^6^.

Calculation of fold change and *p*-value was performed after the normalization scheme described above. Fold change was determined as log 2 (expression ratio). Only miRNAs with a fold change above 0.4 were considered as changed. The *p*-value was calculated by two-sample *t*-test. The target prediction of miRNAs was performed using Targetscan (http://www.targetscan.org/). The expression cluster analysis of miRNAs was done using Cluster3.0. miRNA data have been deposited in the Gene Expression Omnibus under accession number GSE100531.

### EV Co-Cultures

Extracellular vesicles were isolated from cell culture supernatants from anti-CD3/anti-CD28-stimulated PBMCs as described above. Non-stimulated recipient cells (lymphocytes collected after Ficoll-Paque separation and plastic adherence or the cell line U937) were seeded in a 96-well plate at 100,000 cells/well and co-cultured with isolated EVs for 72 h. The cells were analyzed by flow cytometry, using human anti-PD-1 PE (clone: EH12.2H7, BioLegend) and Live/Dead near-IR (Thermo Fischer Scientific), both used in the recommended concentration. Gating was done on live, single cells.

To evaluate EV functionality, HC lymphocytes collected after Ficoll-Paque separation and plastic adherence were pre-stimulated for 72 h. Lymphocytes were washed, and then co-cultured with EVs isolated from RA and HC PBMC cultures, in the presence of plate-bound anti-CD3 and soluble anti-CD28 (both 1 µg/ml). To ensure the presence of a ligand for PD-1, 1 µg/ml soluble rhPD-L1 (R&D systems) was added to the cultures. The supernatant from the last UC of the EVs was used as a negative control. Total cell count was assessed after 72 h using a CCK-8 kit (Sigma-Aldrich). A titration curve with known cell counts was used as a calibrator.

### Mice

All mice were on the C57BL6/J background. PD-1^−/−^ mice were kindly provided by Professor Arlene Sharpe, Harvard Medical School (30). Wild-type (WT) mice were purchased from Janvier, France. Twelve-week-old mice were sacrificed. The spleens were homogenized through a 70 µm filter and washed prior to red blood cell lysis (Sigma-Aldrich). The cells were subsequently washed twice and seeded in a 6-well plate with 30 × 10^6^ cells/well. Cells were stimulated with plate-bound anti-mouse CD3, 1 µg/ml (clone: 145-2C11, BD) and anti-mouse CD28, 2 µg/ml (clone: 37.51, BD) in EV free media. After 72 h of stimulation EVs were collected from WT cells according to the previously described protocol for human cells. The collected EVs were co-cultured with PD-1^−/−^ cells and anti-CD3/anti-CD28 for 48 h. Cells were analyzed by flow cytometry, using anti-mouse PD-1 BV421 (clone: 29F.1A12, Biolegend), -CD3 PerCP-Cy5.5 (clone: 145-2C11), -CD4 PE (clone: GK1.5), -CD8 APC (clone: 53-6.7), -CD19 FITC (clone: ID3) (all from BD), and Live/Dead Aqua fix (Thermo-Fischer). Antibodies were used in the concentrations recommended by the manufacturer. Doublets and dead cells were excluded and gating was done on CD3^+^CD4^+^ T cells.

### Statistics

Data were analyzed as parametric data and presented as mean with SD unless otherwise specified. Differences in data were analyzed using Student’s *t*-test. When applicable, paired *t*-test was used.

## Results

### CD4^+^ T Cells with High Expression of Co-Inhibitory Receptors Are Present in the RA Joint

**Figure 1:**
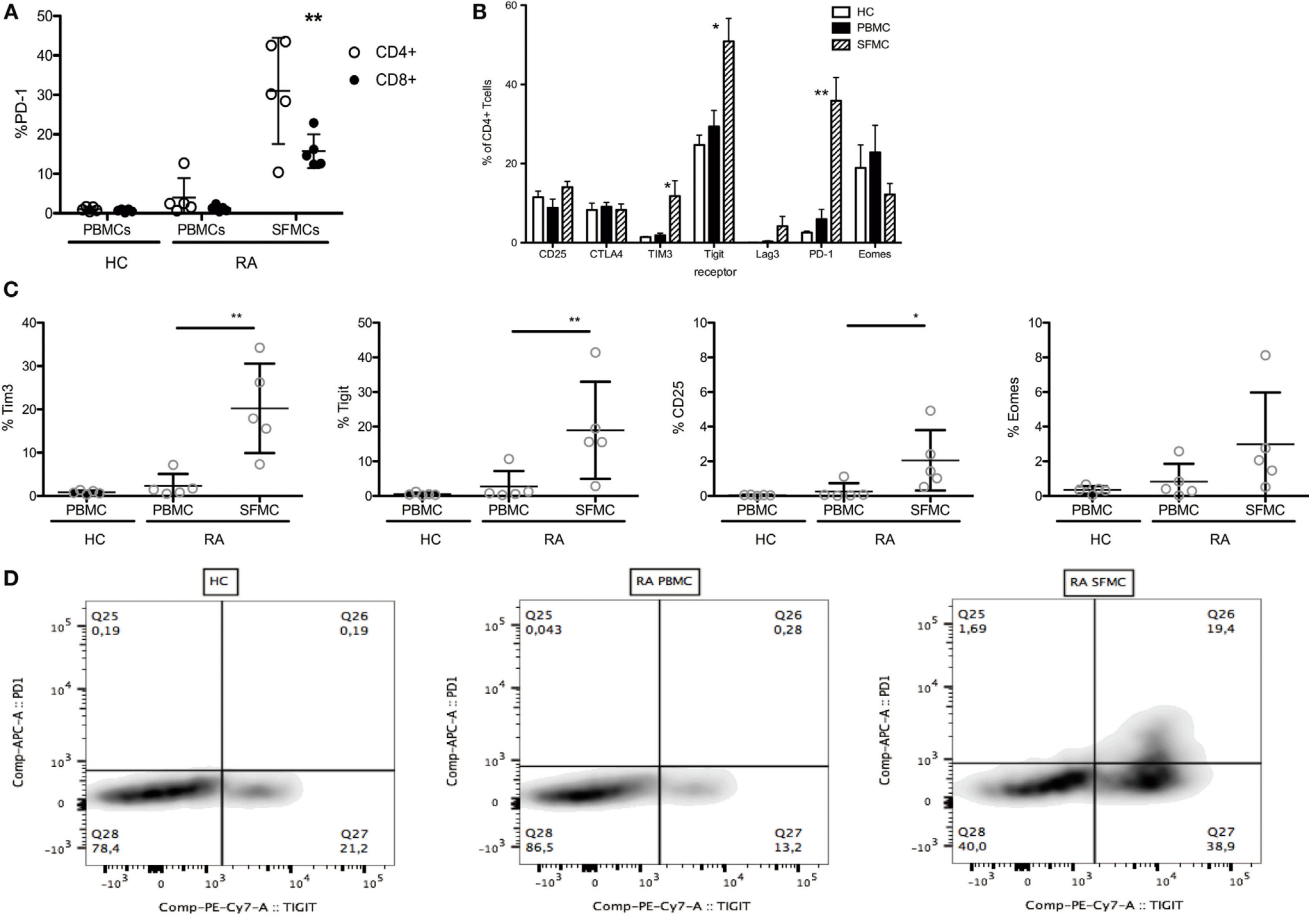
Rheumatoid arthritis (RA) synovial fluid mononuclear cells (SFMCs) express markers of T cell exhaustion. Healthy control (HC) peripheral blood mononuclear cells (PBMCs) (*n* = 5) and paired RA PBMCs and SFMCs (*n* = 5) were stained for their expression of markers associated with T cell exhaustion. **(A)** Expression of PD-1 on CD4^+^ (open circles) and CD8^+^ (closed circles) cells from HC PBMCs and RA PBMCs and SFMCs. PD-1 expression was significantly increased on both CD4^+^ and CD8^+^ cells from RA SFMCs compared to both RA PBMCs and HC PBMCs. **(B)** Surface expression of Tim-3, Tigit, Lag-3, CTLA4, PD-1, and CD25 on CD4^+^ cells from HC PBMCs and paired RA PBMCs and SFMCs. HC: clear bars, PBMCs: filled bars, and SFMCs shaded bars. Expression of Tim-3, Tigit, and PD-1 was significantly increased on RA SFMCs compared to both RA PBMCs and HC PBMCs. **(C)** Staining for co-expression of PD-1 and other markers of T cell inhibition. Gating on CD4^+^ PD-1^+^ cells. Tim3, Tigit, and CD25 was increased in RA SFMCs compared to both RA PBMCs and HC PBMCs. **(D)** Exemplary dot-plots of PD-1/Tigit co-expression on CD4^+^ cells from HCs, PBMCs, and SFMCs (*p < 0.05). Bars represent mean with SD.

We first evaluated the expression of T cell co-inhibitory receptors on CD4^+^ and CD8^+^ T cells from the joints of RA patients. Both CD4^+^ and CD8^+^ SFMCs had increased PD-1 expression compared to both RA PBMCs and HC PBMCs (Figure 1A). In the CD4^+^ cell population, we also investigated expression of other markers associated with T cell inhibition. Here, we observed a significant upregulation of Tim-3 and Tigit on SFMCs (Figure 1B). Furthermore, we also analyzed these receptors on the CD4^+^ PD-1^+^ SFMCs and found them co-expressed with PD-1 (Figures 1C,D).

**Figure 2:**
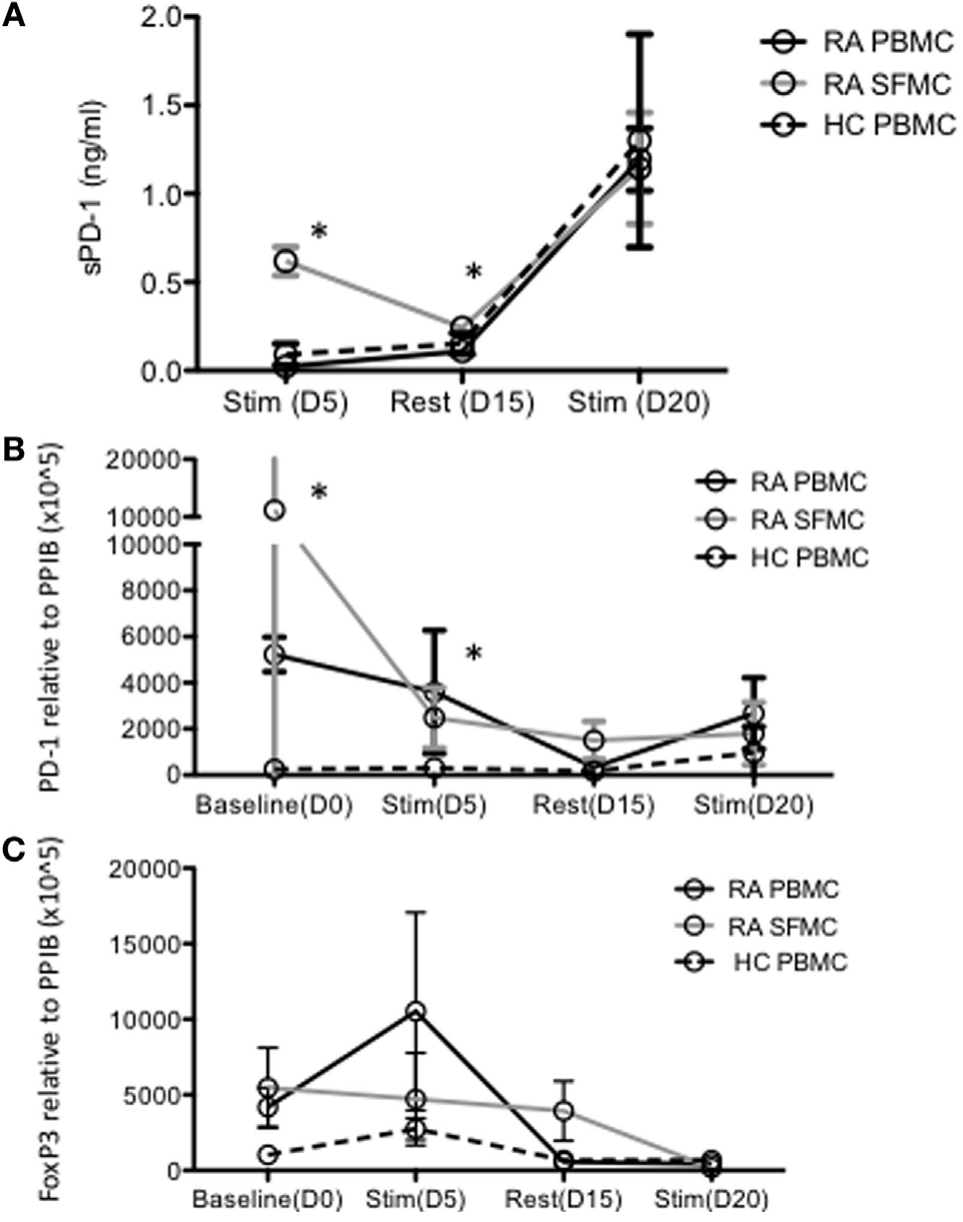
Levels of soluble PD-1 (sPD-1) and corresponding programmed death 1 (PD-1) expression evaluated by qPCR are increased in rheumatoid arthritis (RA) synovial fluid mononuclear cells (SFMCs) in long-term cell culture supernatants. **(A)** Soluble PD-1 in long-term cell cultures determined with ELISA. Soluble PD-1 is increased in RA SFMCs after 5 days of stimulation and additional 10 days rest. After restimulation sPD-1 levels are equivalent in all cultures. **(B)** PD-1 messengerRNA (mRNA) expression assessed by real-time PCR in long-term cell cultures. PD-1 expression was increased at baseline and after 5 days of stimulation in both RA SFMCs and PBMCs compared to healthy control (HC) PBMCs. **(C)** Foxp3 mRNA expression assessed by real-time PCR in long-term cultures. Fox-P3 expression did not differ between the cultures and the expression pattern was different from that of PD-1, suggesting PD-1 not to originate from regulatory T cells. Data were pooled from three different donors, with paired samples of RA PBMCs and SFMCs. Data are presented as mean with SD. Samples compared by two-way ANOVA and one-sample *t*-test. Asterisk (*) represents significant difference, *p* < 0.05.

Culturing HC PBMCs and RA PBMCs and SFMCs under conditions resembling continuous antigen presentation and thereby exhaustion, increased soluble PD-1 (sPD-1) in the cell culture supernatant in all cultures. However, sPD-1 content in PBMC culture supernatants did not reach that of SFMC culture supernatants until day 20 (Figure 2A). Evaluating PD-1 expression by qPCR again revealed a different expression pattern in SFMCs, with the highest expression before stimulations. After continuous stimulations, the PD-1 expression on both HC and RA PBMCs became similar to that of RA SFMCs (Figure 2B). The expression of FoxP3 followed a different pattern upon stimulation and did not differ between the cell types (Figure 2C), suggesting the expression of PD-1 not to be attributed to regulatory T cells.

### Extracellular Vesicles Are Present in RA Plasma and Synovial Fluid and Express PD-1 in RA

**Figure 3:**
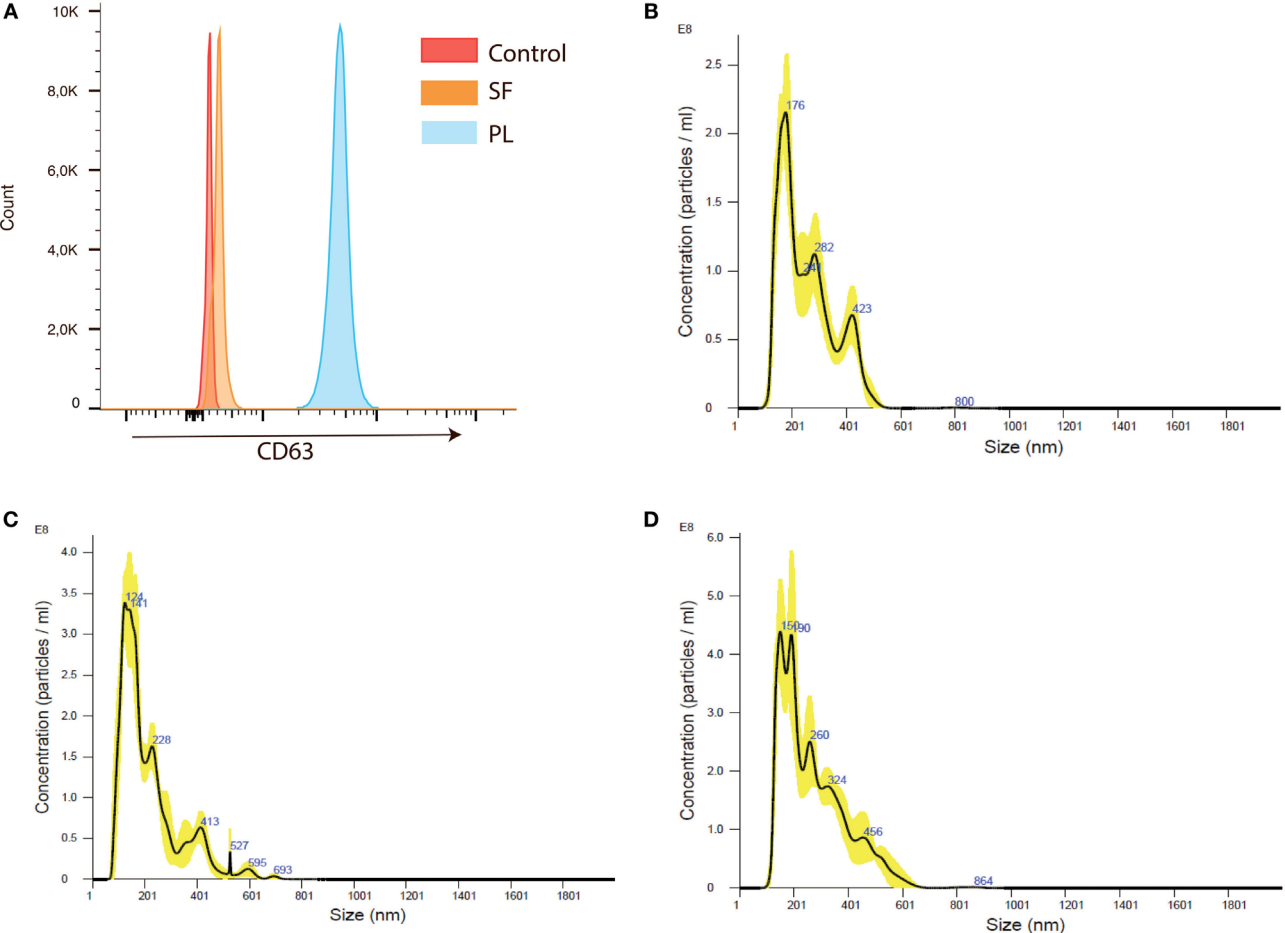
Extracellular vesicles (EVs) are present in rheumatoid arthritis (RA) plasma and synovial fluid and can be isolated from RA peripheral blood mononuclear cells (PBMC) and synovial fluid mononuclear cells (SFMC) cell cultures. **(A)** Extracellular vesicle (EVs) coupled to CD63 positive beads were detected in both plasma and synovial fluid from RA patients. Flow plot is representative of three individual experiments. The presence and size distribution of EVs generated from anti-CD3/anti-CD28-stimulated cell cultures. **(B)** EVs from HC PBMC cultures. **(C)** EVs from RA PBMC cultures and **(D)** EVs from RA SFMC cultures. Representative plot of EVs isolated from cultures from three different HC donors and from four RA patients with paired PBMCs and SFMCs.

The presence of EVs in RA plasma and synovial fluid was confirmed by expression of the EV marker CD63 (31) by flow cytometry (Figure 3A). To obtain a more homogeneous EV fraction, these were isolated from RA and HC cell culture supernatants and their size determined by NanoSight. The EVs ranged in size between 120 nm and 800 nm (Figures 3B–D). The EV size distribution did not differ between the RA and HC cell cultures (not shown).

**Figure 4:**
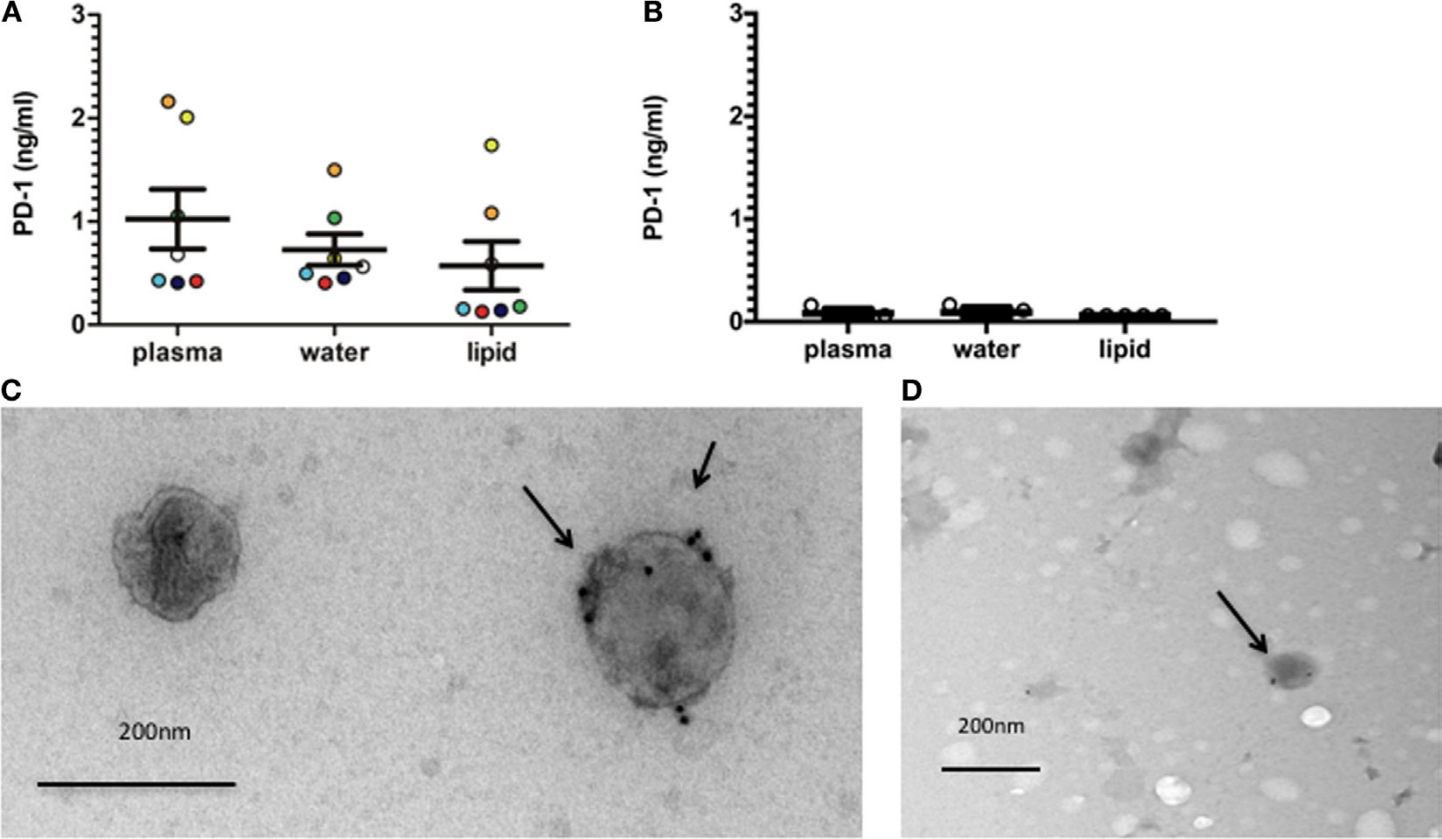
EVs (extracellular vesicles) isolated from RA (rheumatoid arthritis) plasma and RA cell culture supernatants express programmed death 1 (PD-1). **(A)** PD-1 in EVs from RA plasma and synovial fluid isolated by gradient centrifugation, resulting in a hydrophobic and a water-soluble fraction. Each sample is represented with a unique dot-pattern (plasma *n* = 5, synovial fluid: *n* = 2). Bars represent mean ± SEM. **(B)** PD-1 in EVs from HC plasma isolated by gradient centrifugation in accordance with RA samples (*n* = 5). Cut-off of the ELISA was calculated as two SDs of the blank. **(C)** Electron microscopy (EM) image of EVs isolated from cell culture supernatants from RA peripheral blood mononuclear cells (PBMCs) and stained for PD-1 expression (indicated by an arrow). Data are representative of two individual experiments with three different donors. Size indicator: 200 nm. **(D)** EM image of EVs isolated from cell culture supernatants from HC PBMCs and stained for PD-1 expression (indicated by an arrow). Data are representative of two individual experiments. Size indicator: 200 nm.

We have previously reported elevated levels of sPD-1 in RA patients (29). We, therefore, examined the presence of PD-1 in EVs isolated from RA plasma and synovial fluid by a gradient centrifugation. This resulted in a hydrophobic fraction, hypothesized to contain EVs, and a water-soluble fraction that should be EV free. The total amount of sPD-1 in plasma was 1.02 ng/ml ± 0.29 ng/ml. PD-1 was detected in both the lipid fraction (0.57 ng/ml ± 0.24 ng/ml) and the water fraction (0.73 ng/ml ± 0.15 ng/ml) (Figure 4A). In line with previous results (29), we observed very little sPD-1 in HC plasma. sPD-1 was detected in one HC in both plasma and the water-soluble fraction (0.16 ng/ml) and one additional HC in the water-soluble fraction (0.11 ng/ml). PD-1 was not detected in the lipid fraction (Figure 4B). This suggested PD-1 to be present both in a soluble form and in association with EVs in RA patients. The slightly increased total sPD-1 expression combining the two separated fractions could be due to the purification process. EVs that are added concentrated to the ELISA plate would bind quickly, whereas they are more diluted when using plasma. To confirm PD-1 expression in the EVs, we used EVs isolated from cell culture supernatants by a series of ultracentrifugation steps and analyzed these by immunogold EM. PD-1 was found expressed in double lipid bilayer membrane EVs with a size of 120–150 nm (Figures 4C,D). In the supernatant from the cultured cells, we observed expression of PD-1 in EVs from HC and RA PBMCs.

### RA Mononuclear Cell-Derived EVs Incorporate Less miRNA with Potential Targeting Sites in PD-1 and PD-L1 mRNAs

**Figure 5:**
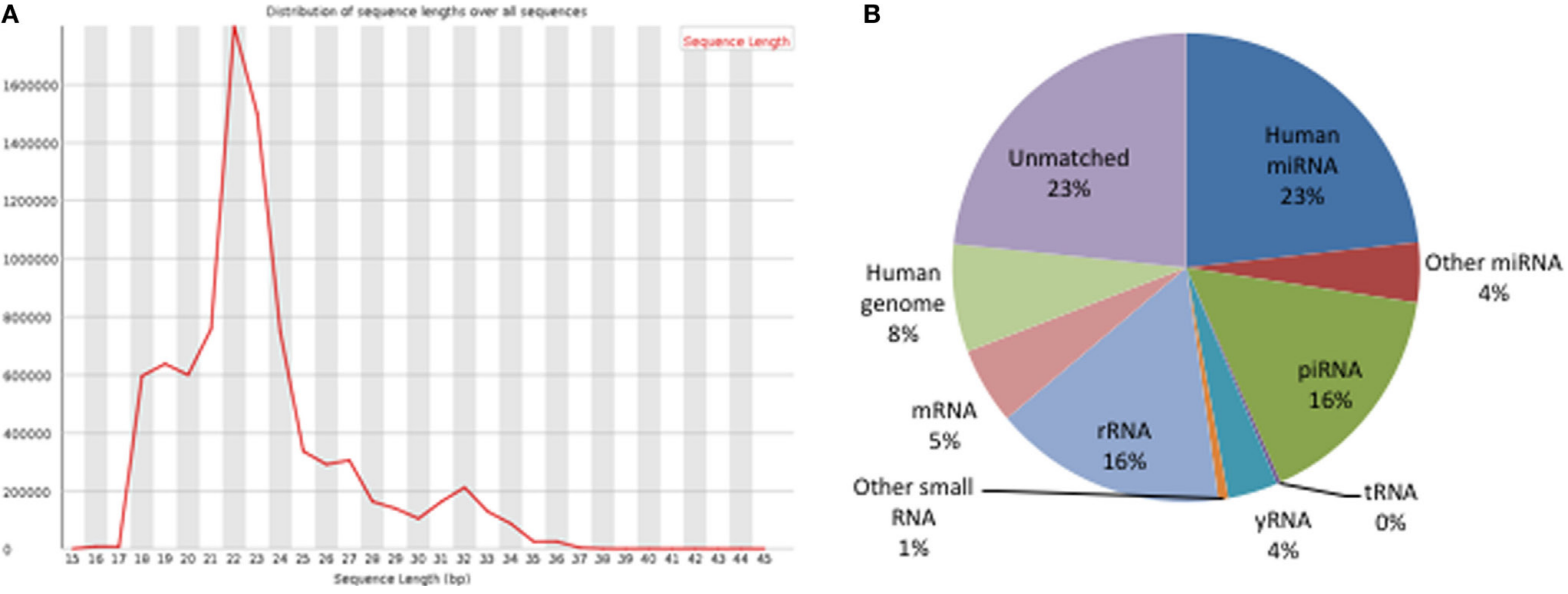
Analysis of small RNA NGS data. **(A)** Length distribution of clean reads: the height of peaks matched with the abundance of reads. **(B)** Annotation of clean reads. The pie chart shows the percentage of different types of small RNAs in the entire sequencing data.

**Figure 6:**
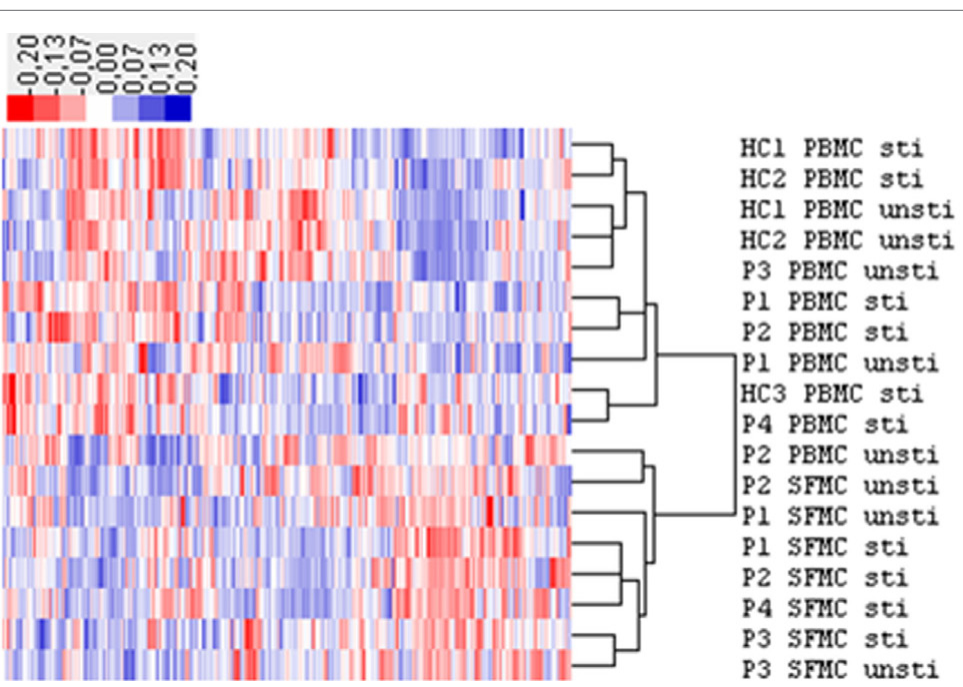
Hierarchical clustering analysis of microRNA (miRNA) expression in extracellular vesicles (EVs) from healthy control (HC) peripheral blood mononuclear cell (PBMC), rheumatoid arthritis (RA) PBMC, and RA synovial fluid mononuclear cells (SFMC). The expression (RPM) of these miRNAs was more than 50 in at least one sample. The expression cluster analysis of human miRNA annotated in all the samples showed a different miRNA expression pattern in EVs from RA SFMCs compared with that of EVs from RA and HC PBMCs. HC PBMCs: stimulated *n* = 3, non-stimulated *n* = 2. RA PBMCs: stimulated *n* = 3, non-stimulated *n* = 3, RA SFMCs; stimulated *n* = 4, non-stimulated *n* = 3. The “*n*” number refers to the samples, where we were able to obtain enough EVs to perform the miRNAseq. Patient samples are paired. RPM, reads per million; P, RA patient; unsti, non-stimulated; sti, stimulated.

**Figure 7:**
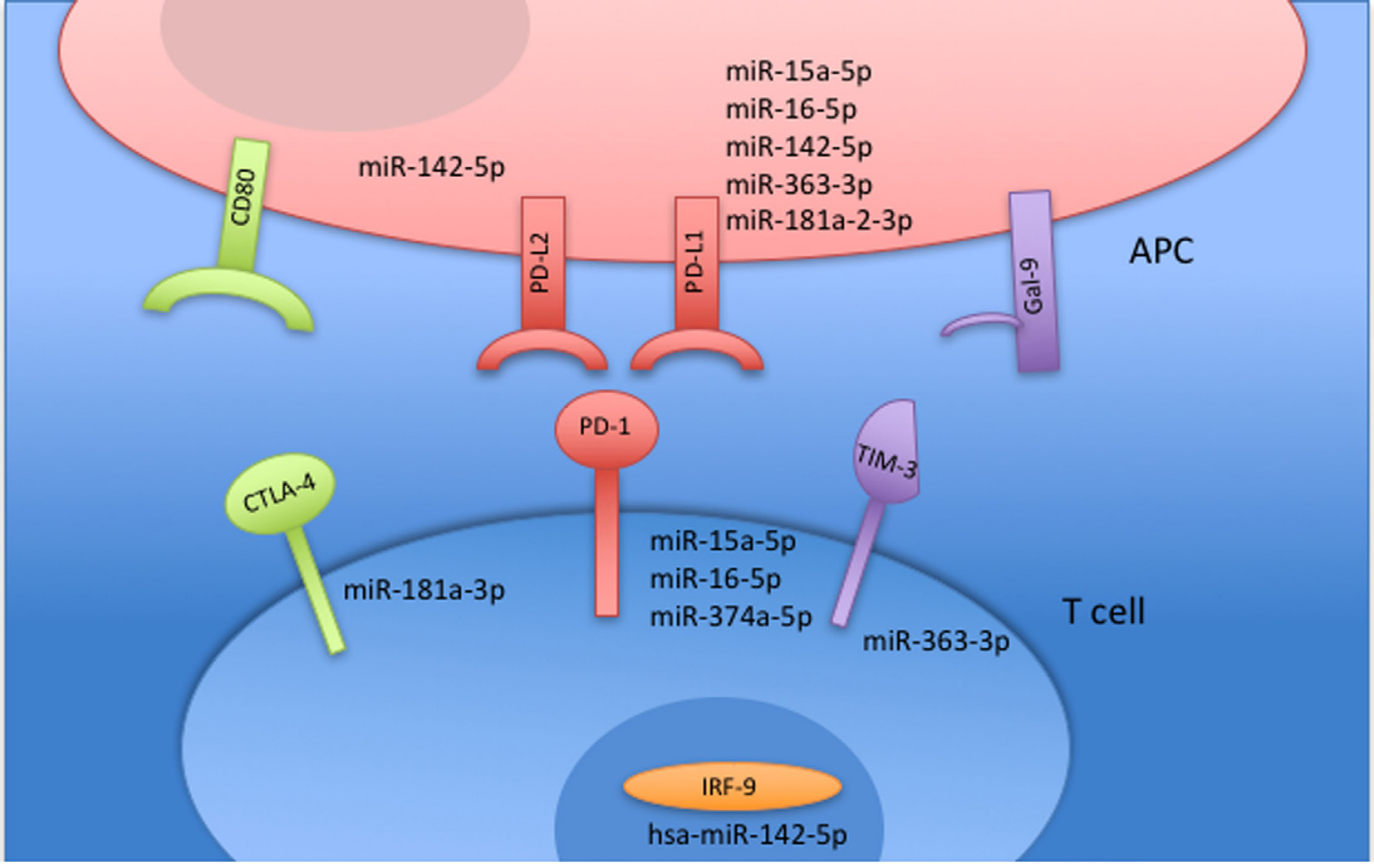
MicroRNAs (miRNAs) targeting receptors associated with T cell exhaustion are downregulated in non-stimulated rheumatoid arthritis (RA) peripheral blood mononuclear cells (PBMCs) compared with non-stimulated healthy control (HC) PBMC. All miRNAs related to T cell inhibition and an exhausted T cell profile were significantly downregulated in EVs from RA PBMCs compared with EVs from HC PBMCs. All miRNAs are downregulated and placed in relation with their associated pathway.

Small RNAs in EVs generated from paired non-stimulated and stimulated samples of RA SFMCs and RA and HC PBMCs were profiled by sequencing to characterize RNAs present in EVs from different cell subsets. Measuring the RNA length distribution of all the samples revealed a peak at 22 nucleotides, consistent with the size of miRNAs (Figure 5). Expression cluster analysis revealed that annotated miRNAs differed in RA SFMC EVs and PBMC EVs (Figure 6). Therefore, we also decided not to compare the miRNA expression directly between EVs from PBMCs and SFMCs. In EVs isolated from non-stimulated PBMCs, there was a significant change in 12 miRNAs when comparing RA with HC [Abs log2(fold change) ≥0.7, *p*-value < 0.05] and 11 of these were downregulated in EV’s from RA PBMCs. The majority of the significantly downregulated miRNAs had putative targets in PD-1/PD-ligands mRNAs (Figure 7; Tables 1 and 2), suggesting that EVs incorporate information to increase immune regulation or potentially promote T cell exhaustion in recipient cells.

**Table 1:** MicroRNAs (miRNAs) predicted to target programmed death 1 (PD-1) or its ligands, TIM-3, Cytotoxic T-lymphocyte-associated protein 4 (CTLA-4) or IRF9 downregulated in non-stimulated rheumatoid arthritis (RA) peripheral blood mononuclear cell (PBMC) compared with non-stimulated healthy control (HC) PBMC.

Name	Log2 fold change	*p*-Value
**PD-1**		
hsa-miR-16-5p	−1,278	0.0064
hsa-miR-15a-5p	−1,309	0.015
hsa-miR-374a-5p	−2,823	0.027
**PD-L1**		
hsa-miR-181a-2-3p	−0,7283	0.032
hsa-miR-363-3p	−0,9262	0.028
hsa-miR-142-5p	−1,064	0.030
hsa-miR-16-5p	−1,278	0.0064
hsa-miR-15a-5p	−1,309	0.015
**PD-L2**		
hsa-miR-142-5p	−1,064	0.030
**TIM-3**		
hsa-miR-363-3p	−0,9262	0.028
**IRF-9**		
hsa-miR-142-5p	−1,064	0.030
**CTLA-4**		
hsa-miR-181a-3p	−0,7780	0.035

*All miRNAs related for these coinhibitory molecules were significantly downregulated in extracellular vesicles (EVs) from RA PBMCs compared with EVs form HC PBMCs (HC, *n* = 2; RA, *n* = 3). Samples were compared by a two-sample *t*-test. *p* < 0.05 is considered statistically significant*.

**Table 2:** MicroRNAs (miRNAs) changed in non-stimulated rheumatoid arthritis (RA) peripheral blood mononuclear cells (PBMC) compared with non-stimulated healthy control (HC) PBMC.

miRNA name	HC1 reads per million (RPM)	HC2 RPM	HC mean	Patient 1 RPM	Patient 2 RPM	Patient 3 RPM	Patient mean	Fold change	*p*-Value
hsa-miR-1246	774	283	529	1,332	1,667	1,347	1,449	1.45	0.028
hsa-miR-181a-2-3p	963	797	880	434	546	614	531	−0.73	0.031
hsa-miR-181a-3p	432	348	390	262	181	239	227	−0.78	0.035
hsa-miR-30d-5p	2,332	2,442	2,387	832	1,555	1,553	1,313	−0.86	0.041
hsa-miR-363-3p	384	301	342	202	139	199	180	−0.93	0.028
hsa-miR-30e-5p	570	456	513	232	283	284	267	−0.94	0.014
hsa-miR-142-5p	7,704	9,701	8,703	4,527	2,818	5,143	4,163	−1.06	0.030
hsa-miR-181a-5p	113,294	125,404	119,349	46,381	57,891	47,506	50,593	−1.24	0.001
hsa-miR-16-5p	9,094	9,990	9,542	2,912	3,979	4,916	3,936	−1.28	0.006
hsa-miR-15a-5p	1,367	1,356	1,362	557	330	761	549	−1.31	0.015
hsa-miR-23a-3p	631	632	632	194	179	391	255	−1.31	0.023
hsa-miR-29a-3p	2,056	1,165	1,610	578	583	617	593	−1.44	0.054
hsa-miR-29c-3p	416	206	311	28	46	96	57	−2.46	0.054
hsa-miR-374a-5p	64	106	85	12	0	24	12	−2.82	0.026

*miRNAs changed in EVs from RA PBMCs compared to HC PBMCs. Most miRNAs were downregulated and only one was upregulated. The search was guided by fold change >log0.4 and samples were compared by a two-sample t-test. p < 0.05 is considered statistically significant*.

### Stimulation Results in Different miRNA Profiles in EVs from RA PBMC, RA SFMC, and HC PBMC

The effect of stimulation on the incorporation of miRNAs in EVs was assessed. Here, the miRNA content in EVs from RA SFMCs differed significantly from that of both RA and HC EV PBMCs. Stimulation changed expression [Abs (fold change) ≥0.4] of 31 miRNAs in EVs from RA SFMCs, 69 miRNAs in EVs from RA PBMCs, and 57 miRNAs in EVs from HC PBMCs. Generally, stimulation caused upregulation of miRNAs in the EVs. We investigated if miRNAs changed by stimulation were predicted to function in the PD-1 pathway and other co-inhibitory pathways. We observed miRNAs targeting PD-1 and its two ligands PD-L1 and PD-L2 and, in addition, TIM3, CTLA4, and IRF9 (a transcription factor that promotes expression of the *PDCD1 gene*). The largest changes in EV miRNA content were related to PD-1. In EVs from RA PBMCs and HC PBMCs, respectively, 9/10 and 7/10 miRNAs targeting PD-1 were upregulated upon stimulation. By contrast, only four PD-1 related miRNAs were changed in the EVs generated from stimulation of RA SFMCs, and of these only two were upregulated (Table 3). A similar picture emerged regarding PD-L1 and PD-L2 (Table 3). This suggests that EVs from PBMCs, upon stimulation, incorporate more miRNAs repressing expression of PD-1, its ligands, and other co-inhibitory receptors, whereas EVs from RA SFMCs fail to upregulate these miRNAs. Comparing the miRNA content between stimulated RA EVs and HC EVs, some miRNAs targeting the PD-1 pathway were still significantly downregulated in RA EVs.

**Table 3:** MicroRNAs (miRNAs) related to co-inhibitory receptors are upregulated in extracellular vesicles (EVs) following stimulation.

miRNA target	EVs from	Total number of changed miRNAs	Upregulated miRNAs	Downregulated miRNAs
Programmed death 1 (PD-1)	HC PBMC	10	7	3
	RA PBMC	10	9	1
	RA SFMC	4	2	2
PD-L1	HC PBMC	9	8	1
	RA PBMC	15	13	2
	RA SFMC	8	7	1
PD-L2	HC PBMC	15	12	3
	RA PBMC	8	7	1
	RA SFMC	4	3	1
TIM3	HC PBMC	6	5	1
	RA PBMC	9	9	0
	RA SFMC	6	5	1
CTLA4	HC PBMC	3	3	0
	RA PBMC	5	5	0
	RA SFMC	2	2	0
IRF9	HC PBMC	2	1	1
	RA PBMC	1	1	0
	RA SFMC	2	2	0

*Changes in miRNAs in EVs after cell culture stimulation are shown. miRNAs related to PD-1 and PD-L2 were upregulated in EVs from both HC PBMCs and RA PBMCs but not from RA SFMCs*.

### PD-1 Is Transferred by EVs to Co-Cultured Cells

**Figure 8:**
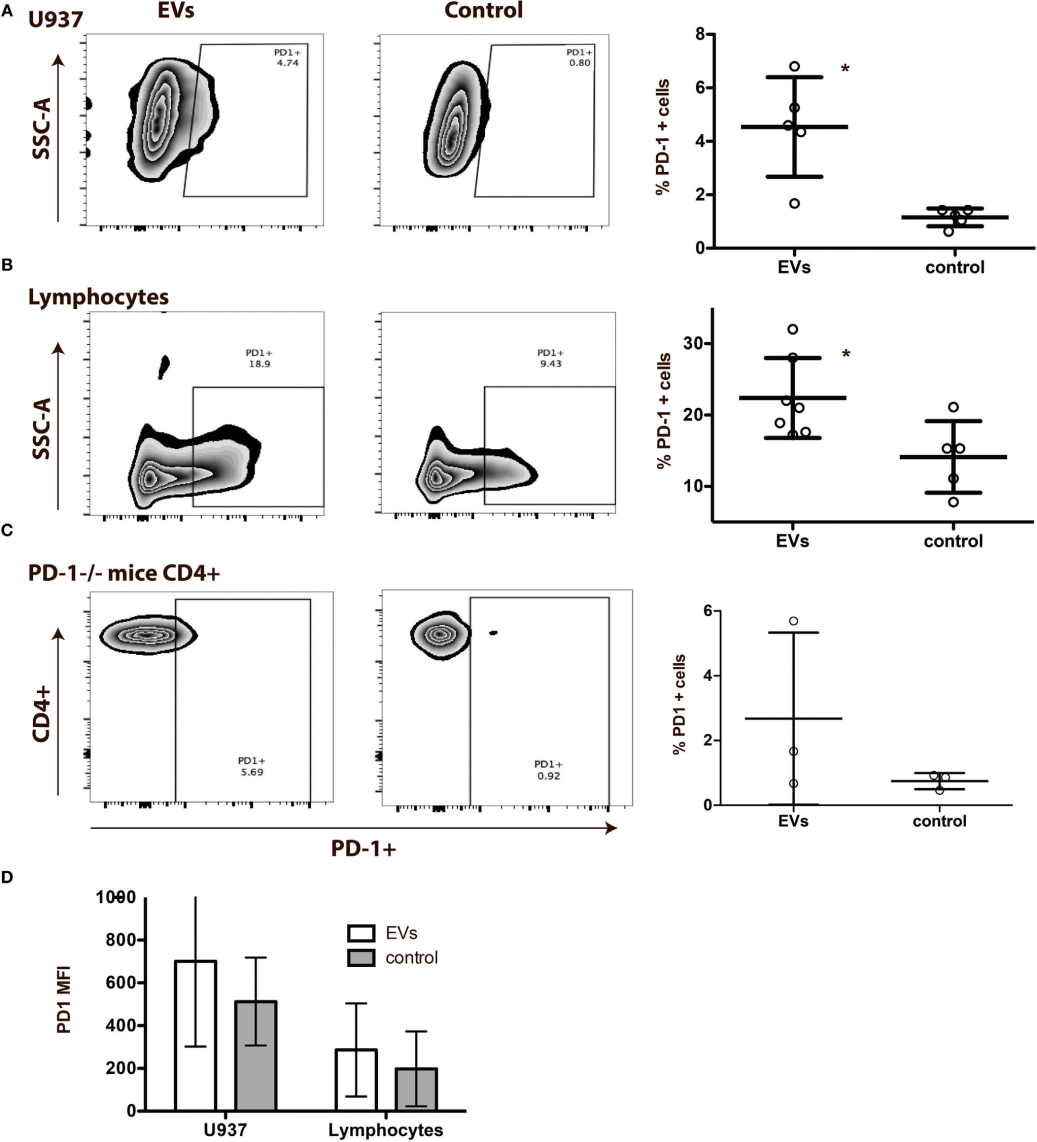
Programmed death 1 (PD-1) from extracellular vesicles (EVs) is transferred to other cells in an *in vitro* system. **(A)** EVs isolated from peripheral blood mononuclear cells (PBMC) cell culture supernatants in co-culture with the PD-1 negative cell line U937 for 72 h. Representative flow plots of control U937 cells cultured with EVs (left), controls without EVs (right) and summarized PD-1 expression on U937 cultured with and without (control) EVs (*N* = 5). Asterisks (**) indicates *p* < 0.01. Error bars represent mean ± SD. **(B)** EVs isolated from PBMC cell culture supernatants in co-culture with lymphocytes for 72 h. Representative flow plots of lymphocytes cultured with EVs (left), control lymphocytes without EVs (right), and summarized PD-1 expression on lymphocytes cultured with, and without (control) EVs (*N* = 5). Asterisks (*) indicates *p* < 0.05. Error bars represent mean ± SD. **(C)** EVs isolated from cell culture supernatants from wild-type (WT) mice spleen cells in co-culture with spleen cells from PD-1^−/−^ mice for 48 h. Representative flow plots of PD-1^−/−^ cells cultured with EVs from WT mice (left), control PD-1^−/−^ cells (right) without EVs, and summarized PD-1 expression on PD-1^−/−^ cells cultured with and without WT EVs (*N* = 3). Error bars represent mean ± SD. **(D)** Corresponding MFI values of PD-1 on U937 and lymphocyte after co-culture with EVs. Data are presented as mean ± SD.

In consideration of the close relationship of the miRNA profiles in the EVs with the PD-1 pathway, we examined whether the PD-1 receptor could be transferred to other cells after co-culturing of the cells with EVs. First, we used a cell line, U937, which we confirmed negative for PD-1 expression by qPCR and flow cytometry. A PD-1 expression of 4.0% (1.7−6.3%) was observed on U937 cells after co-culturing with EVs (Figure 8A). Next, we examined the effect of co-culturing lymphocytes with EVs and observed an increase in PD-1 expression from 14.1% (7.9−20.4%) to 22.4% (17.2−27.6%) (Figure 8B). These increases in PD-1 were followed by an increase in the median fluorescence intensity (MFI) (Figure 8D). Number of live cells did not differ between the cultures (not shown).

To validate the potential of EVs to transfer PD-1 to recipient cells, we co-cultured spleen lymphocytes from PD-1^−/−^ mice with EVs isolated from spleen cells of WT mice. After exposure to EVs from the WT mice, we observed PD-1 expression on the PD-1^−/−^ cells, on up to 6% of both CD4^+^ cells (Figure 8C) and CD19^+^ cells (data not shown). The MFI of PD-1 on CD4^+^ cells from PD-1^−/−^ mice increased from 38.5 (38.5–46) to 73.2 (69.4–79.6). These data support that EVs transfer the co-inhibitory receptor PD-1 to cells in the microenvironment.

### EVs from RA PBMC Cultures Increase Proliferation in Recipient Lymphocytes

**Figure 9:**
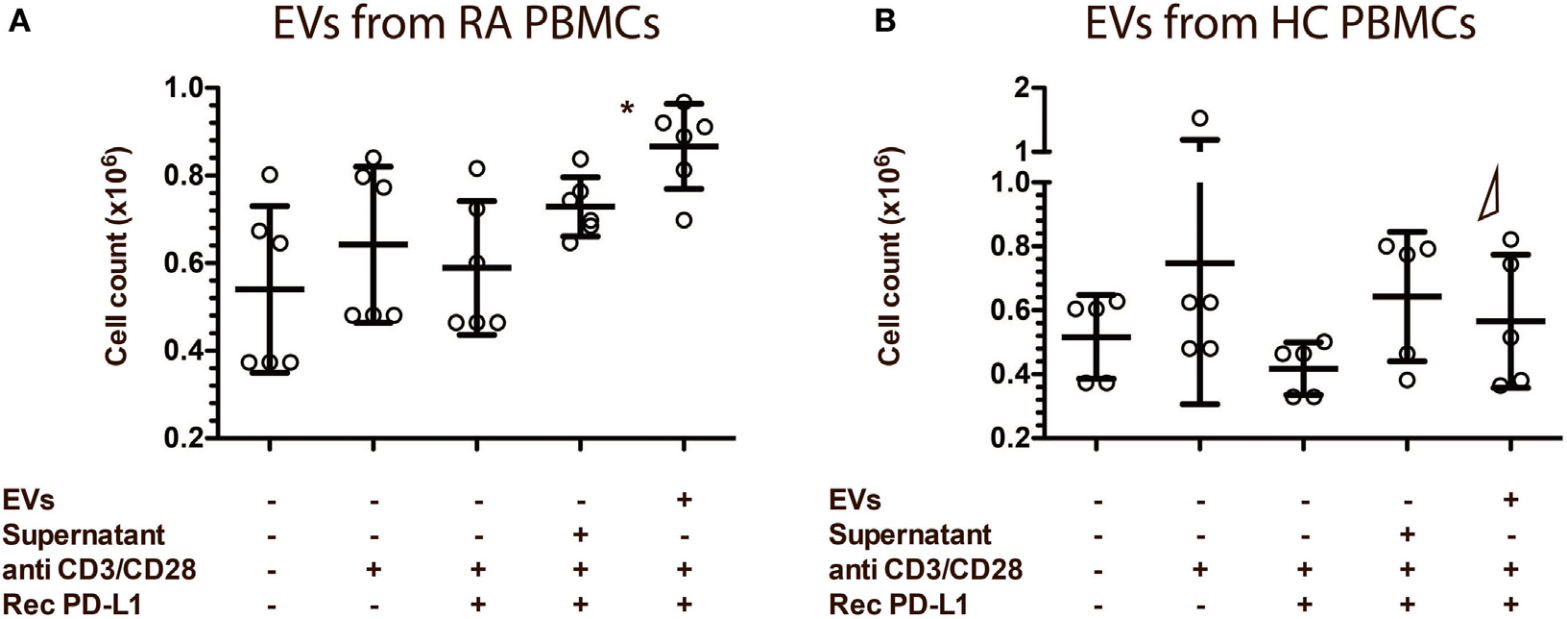
Extracellular vesicles (EVs) from rheumatoid arthritis (RA) patients increase recipients cell proliferation in lymphocyte co-cultures. EVs isolated from peripheral blood mononuclear cell (PBMC) cell culture supernatants. RA EVs (*n* = 6) **(A)** and healthy control (HC) EVs (*n* = 5) **(B)** co-cultured with lymphocytes. Lymphocytes were from HC donors, different from the ones used to collect the EVs. EV/PBMC cultures were re-stimulated with anti-CD3/anti-CD28 in the presence of rhPD-L1 (1 µg/ml). Asterisks (*) indicates *p* < 0.05. Δ represents a significant difference (*p* < 0.05) to the corresponding experiment with EVs from RA PBMCs. Error bars represent mean ± SD.

To determine the functional capacity of the EVs isolated from both RA and HC PBMC cultures, we evaluated lymphocyte count in an assay where EVs and lymphocytes were co-cultured. To ensure the presence of a ligand for PD-1, we added rhPD-L1 to the culture. Lymphocytes co-cultured with EVs from RA PBMCs proved to be in a significantly higher number than lymphocytes co-cultured with the control, but also than lymphocytes co-cultured with EVs from HC PBMCs (Figure 9A). EVs from HC PBMCs did not affect numbers of lymphocytes in the culture (Figure 9B). This could indicate that RA EVs increase either lymphocyte proliferation or survival and suggests a stimulatory capacity of RA EVs, and that the PD-1 transferred to the cells by the EVs is not sufficient to eliminate the stimulation.

## Discussion

In this study, we identified for the first time PD-1 expression by EVs and owing to the suggested role of this protein and EVs in RA pathogenesis (32–35), we further characterized the PD-1 expression in EVs in RA patients. Exhausted T cells are associated with chronic inflammatory diseases, including RA (36). Here, we confirm the presence of T cells that co-express PD-1, and other receptors associated with T-cell exhaustion in the RA joint.

Cells can only be regarded as truly exhausted, when they also present with reduced functions, being decreased cytokine production or proliferation. However, reduced T-cell functions in RA SFMCs were already described years ago (37, 38). Based on these early data on RA SFMCs and our recent findings characterizing the high expression of co-inhibitory receptors on the T cells, the RA joint could be regarded as a microenvironment favoring T cell exhaustion.

Programmed death 1 is a pivotal marker of exhausted T cells, besides being an essential co-inhibitory receptor. It is crucial in maintaining peripheral tolerance, but is also associated with cancer development, chronic infections, and autoimmune diseases (39). We find that PD-1 is not only expressed by EVs, but cells cultured with PD-1 expressing EVs also increase their PD-1 expression. In the context of RA, this cell-to-cell transfer of an inhibitory receptor could be an attempt to control the increased immune activity. We showed that only few cells were affected by EV-transferred PD-1. This finding suggests that in order for the EVs to affect the biological outcome by transferring PD-1, the process has to take place over a prolong period of time and, furthermore, cells presumably have to be in close proximity. The inflamed RA joint may create such an environment. However, it still remains to be elucidated to what degree PD-1 is transferred as a functional receptor. Furthermore, we cannot rule out that this process also takes place in HC, but considering we do not detect PD-1 on EVs from HC plasma this seems less likely.

If RA is left untreated, it will progress into a chronic state with uncontrolled inflammation. Thus, the functional impact of the transferred PD-1 *in vivo* could be questioned. We investigated the functionality in an *in vitro* system and observed that lymphocytes co-cultured with EVs from RA PBMCs proliferated more than both control lymphocytes and lymphocytes co-cultured with EVs from HC PBMCs. This suggests that EVs from RA PBMCs differ significantly from those of HC PBMCs, and that these RA EVs are important contributors in maintaining the disease in a chronic state, despite incorporating information related to a co-inhibitory receptor.

Based on our results, it could be hypothesized that the transferred PD-1 exerts an antagonistic effect, or simply that the transferred PD-1 is not sufficient to control the activation induced by the EVs (22). Also, this supports that EVs from RA patients differ from those of HCs and might be involved in promoting chronicity of the disease. However, some of the processes, e.g. miRNA inhibition, would potentially take place over a longer period of time than the present setup enables (40). We examined miRNA expression in EVs from both RA and HC cell cultures. When studying the miRNA profile from stimulated cell cultures, we observed an upregulation of miRNAs associated with PD-1 and its ligands, as well as other co-inhibitory receptors, including TIM-3 and CTLA-4, both also associated with the exhausted T cell profile. Upon stimulation, EVs showed no major differences in miRNAs when purified from RA and HC PBMCs. However, when we examined RA synovial fluid cultures, these cells did not upregulate miRNAs in the EVs to the same extend. This supports the idea that these cells are “more” exhausted (6, 37, 41, 42) and, therefore, unable to transfer information to shut down co-inhibitory signals in nearby cells. RA synovial fluid cells also exerted a different expression pattern in response to a prolonged stimulation than both HC and RA PBMCs. This is consistent with the microenvironment of the joint being the major site of disease progression and potential development of chronicity.

MicroRNAs are known as important regulators of immune activity in inflammation and are associated with disease activity in both cancers and autoimmunity (25, 26). The miRNA profiles of EVs from non-stimulated PBMCs from RA and HC differed. Both miRNAs related to PD-1 and its ligands, in addition to TIM-3 and CTLA-4 were downregulated. To obtain EVs from especially the non-stimulated cell cultures, we had to use large quantities of human cells. This naturally limits the number of patients that can participate in such a setup. However, despite this we do find significant differences in the miRNA expression profiles among the participants. Thus, EVs from the peripheral circulation in RA patients carry information to promote T cell inhibition and potentially also exhaustion in the steady-state of inflammation. However, it is not sufficient to control disease progression, and it is possible that the exhausted profile also may preserve inflammation in the local environment (38).

We hypothesized that EVs could contribute to the progression of disease in the RA microenvironment and, in conclusion, we suggest that EVs from RA patients transport information related to T cell inhibition and in conjunction, T cell exhaustion. Especially the PD-1 pathway, and that the receptor PD-1 itself is incorporated in the EVs and transferred to the recipient cells. This suggests that EVs from the chronic inflammatory environment carry information that influence the development of chronicity, and that EVs in RA play a central role in disease progression. If chronicity “spreads” in protected transport vesicles, these EVs could potentially be identified as new treatment targets, applicable to all chronic inflammatory diseases.

## Ethics Statement

No animals or humans were directly involved in the study, but cells from both human and euthanized mice were used for the study. Studies on mice were approved by the Danish Animal Experience Inspectorate, protocol number: 2014−15−0201−00001. Studies involving human cells were performed on material from the INART biobank, a study approved by the Danish Data Protection agency and the Ethical Committee, protocol number: 20 121 329.

## Author Contributions

SG conducted most of the experimental work, data analysis, and interpretation, and drafted and edited the manuscript. YY conducted parts of the experimental work, including data analysis, and drafted sections of the manuscript. AH conducted parts of the experimental work, including data analysis, and drafted one part of the manuscript. MV conducted calculations and interpretations of the miRNA data and revised the manuscript. JN conducted the electron microscopy and revised the manuscript. SM, MH, GF, JK, and BD planned and supervised the project, interpreted data and conducted several critical revisions of the manuscript. All authors read and approved the final manuscript, and agreed to be accountable for all aspects of the work.

## Conflict of Interest Statement

The authors declare that the research was conducted in the absence of any commercial or financial relationships that could be construed as a potential conflict of interest.
